# Nitrogen Deposition to and Cycling in a Deciduous Forest

**DOI:** 10.1100/tsw.2001.372

**Published:** 2001-12-05

**Authors:** Sara C. Pryor, Rebecca J. Barthelmie, Margaret Carreiro, Melissa L. Davis, Anne Hartley, Bjame Jensen, Andrew Oliphant, Melissa J. C. Randolph, Justin T. Schoof

**Affiliations:** Department of Geography, Indiana University, Bloomington 47405, USA

**Keywords:** atmospheric fluxes, nitrogen-carbon linkages, temperate forests

## Abstract

The project described here seeks to answer questions regarding the role increased nitrogen (N) deposition is playing in enhanced carbon (C) sequestration in temperate mid-latitude forests, using detailed measurements from an AmeriFlux tower in southern Indiana (Morgan-Monroe State Forest, or MMSF). The measurements indicate an average atmosphere-surface N flux of approximately 6 mg-N m(-2) day(-1) during the 2000 growing season, with approximately 40% coming from dry deposition of ammonia (NH3), nitric acid (HNO3), and particle-bound N. Wet deposition and throughfall measurements indicate significant canopy uptake of N (particularly NH4+) at the site, leading to a net canopy exchange (NCE) of -6 kg-N ha(-1) for the growing season. These data are used in combination with data on the aboveground C:N ratio, litterfall flux, and soil net N mineralization rates to indicate the level of potential perturbation of C sequestration at this site.

## THE ROLE OF NITROGEN IN THE GLOBAL CARBON CYCLE

Although there are approximately 5 billion metric tons of nitrogen (N) in the atmosphere, oceans, terrestrial and marine biota, soil organic matter, and sedimentary rock, less than 2% is bioavailable. N availability is frequently observed to limit primary productivity and hence carbon (C) uptake and storage in a wide array of ecosystems[1,2]. Anthropogenic activity (e.g., combustion of fossil fuels, animal husbandry, and production of fertilizers) has doubled the supply of reactive N to terrestrial ecosystems relative to "natural background"[3]. Temperate deciduous forests contain 11% of biospheric plant mass[4] and hence comprise a significant C store. Although there is controversy regarding the quantitative role that increased N deposition is playing in determining the C sink strength of temperate forests[5,6], it has been proposed that increased N deposition may have been responsible for 0.2 to 1.0 Gt C year$^{-1}$ increased C storage during the 1980s[7].

In terrestrial ecosystems not significantly impacted by anthropogenic emissions, the majority of the required N is supplied by mineralization of nutrients from decomposing organic matter[4] (although direct uptake of organic N as amino acids may make a nonnegligible contribution[8]) with uptake via plant roots. However, atmospheric N inputs to terrestrial ecosystems can constitute an important fraction of a system's total annual N budget and importantly are transferred via the canopy[9].

The research presented here is part of a comprehensive study designed to quantify N pools and fluxes and elucidate links between increased atmosphere-surface N flux and ecosystem response in a temperate deciduous forest.

## SAMPLING LOCATION

Morgan-Monroe State Forest (MMSF) is located at 39°53′N, 86°25′ W in south-central Indiana, U.S., just south of the limit of the late Wisconsin glaciation, and is dominated by ridge/ravine topography. Soils are well-drained inceptisols, formed in residuum from sandstone, siltstone, and shale. The region is covered primarily by secondary successional broadleaf forests located within the maple-beech to oak-hickory transition zone of the Eastern Deciduous Forest. In MMSF, 29 tree species occur; dominant species (about 75% of total basal area) are sugar maple (*Acer saccharum*), tulip poplar (*Liriodendron tulipifera*), sassafras (*Sassafras albidum*), white oak (*Quercus alba*), and black oak (*Q. velutina*). Common understory species include pawpaw (*Asimina triloba*), spicebush (*Lindera benzoin*), and sweet cicely (*Osmorhiza claytonii*), as well as seedlings and saplings of dominant tree species. Most of Indiana was deforested by the early 1900s, but afforestation has occurred in much of central and southern Indiana following abandonment of marginally productive agricultural land. Timber harvests have not occurred in the source area of the flux tower recently, except in one small seed-tree area. The flux tower is 46 m high and extends above a forest canopy that is approximately 25 m high.

The MMSF site is part of the AmeriFlux network established to enhance understanding of C fluxes, net ecosystem production (NEP), and C sequestration in the terrestrial biosphere. C pool sizes and fluxes at MMSF are being measured using ecological and micrometeorological techniques. The eddy correlation derived estimate of annual NEP for the 1998–1999 season is 2.4 t C ha$^{-1}$ year$^{-1}$ (±10%)[10], while the estimate derived from ecological measurements[11] is 2.6 t C ha$^{-1}$ year$^{-1}$.

## NITROGEN FLUX AND POOL MEASUREMENTS AT MMSF

Deposition of atmospheric N to terrestrial ecosystems occurs primarily as a result of wet deposition of $NH_4^+$ and $NO_3^-$, and dry deposition of inorganic gases — principally ammonia ($NH_3$), oxides of nitrogen (NOx), and nitric acid ($HNO_3$) — and N-containing particles, although dissolved organic nitrogen (DON) is known to be a significant component of N in wet deposition and throughfall[12] and is present in the particle phase. As described below, to estimate the atmospheric N flux to the MMSF ecosystem we are conducting measurements of the inorganic fluxes using micrometeorological techniques for the dry deposition fluxes and bulk sampling for wet deposition, throughfall, and stemflow. Instrumentation pertaining to atmospheric measurements of N compounds at MMSF is summarized in Figs. 1 and 2 and Table 1.

To measure wet deposition fluxes, an Aerochem wet/dry collector identical to those used in the NADP network[13] is mounted on the MMSF tower at 26.8 m, and a second system is located 50 m east of the tower under a mixed canopy to measure throughfall composition. Samples are collected weekly and analyzed on a Dionex-120 HPIC. Fluxes are calculated using a precipitation gauge mounted at 46 m on the tower for wet deposition, and the average liquid flux is calculated from a below-canopy network of three gauges for the throughfall fluxes. Previous research has demonstrated enhanced pollutant concentrations in the initial phase of precipitation events and in events characterized by low precipitation amounts, and wet deposition fluxes obtained via operation of Aerochem samplers are subject to uncertainties due principally to undersampling of low precipitation events and delay in sampling onset[14]. The error introduced by these uncertainties has not been quantified at the MMSF site, but the precipitation regime is not characterized by a high number of low-liquid water events, and hence it is assumed that the error is small relative to the spatial variability and the analytical uncertainty of the concentration measurements.

Throughfall chemistry is known to be highly spatially variable and dependent on canopy structure and characteristics[15]. Accordingly, we deploy 18 distributed samplers in clusters of three under, respectively, one pine canopy, two canopies dominated by sugar maples, and three mixed canopies (Table 2). Although most water that penetrates the canopy does so as throughfall, previous research in deciduous forests has indicated stemflow may contribute up to 24% of the N flux to the forest floor[16]. Hence, beginning in summer 2001, limited stemflow observations were added to the measurement suite.

While wet deposition, throughfall, and stemflow are comparatively straightforward to measure, dry deposition of gases and particles is time consuming and expensive to undertake. Hence, most of the dry deposition measurements (Table 1) are being undertaken in intensive field campaigns. Although eddy correlation and eddy accumulation techniques are theoretically superior methods for determining dry deposition fluxes, due to the high demands on instrument technology it is still common, as in this research, to use gradient techniques for some gases and particles. Time-integrated concentrations of $NH_3$ and $HNO_3$ have been measured using active oxalic acid– and NaCl-coated denuders with analysis by HPIC, while continuous measurements of $NH_3$ have been made using WEDD systems. WEDDs use a similar concept to denuders, but water is used to collect the gas of interest and concentrations are measured *in situ*[17]. Three techniques have been used to calculate atmosphere-surface exchange of $HNO_3$ and $NH_3$: the Bowen ratio technique[18,19], a technique based on $C_*$ (the vertical concentration gradient) and

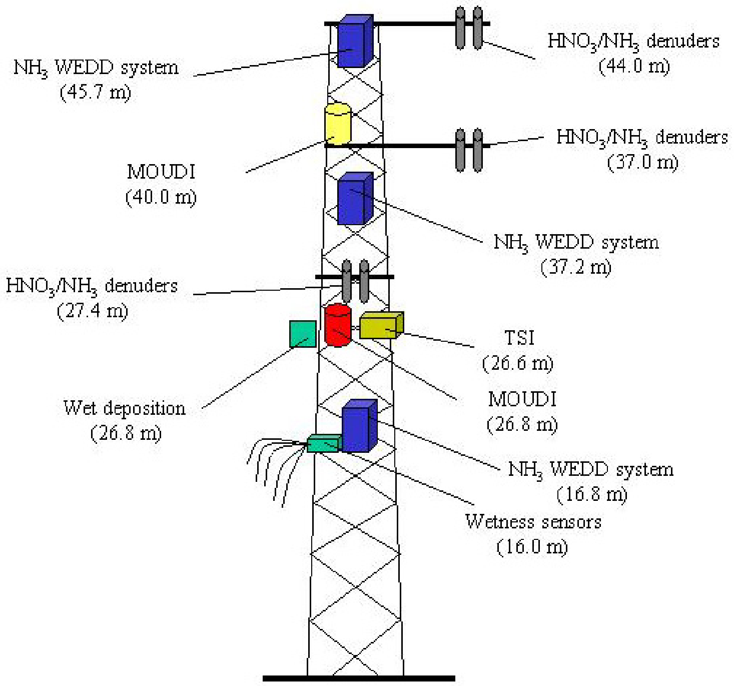

**FIGURE 1.** Instrumentation deployed at MMSF during episodic field experiments.

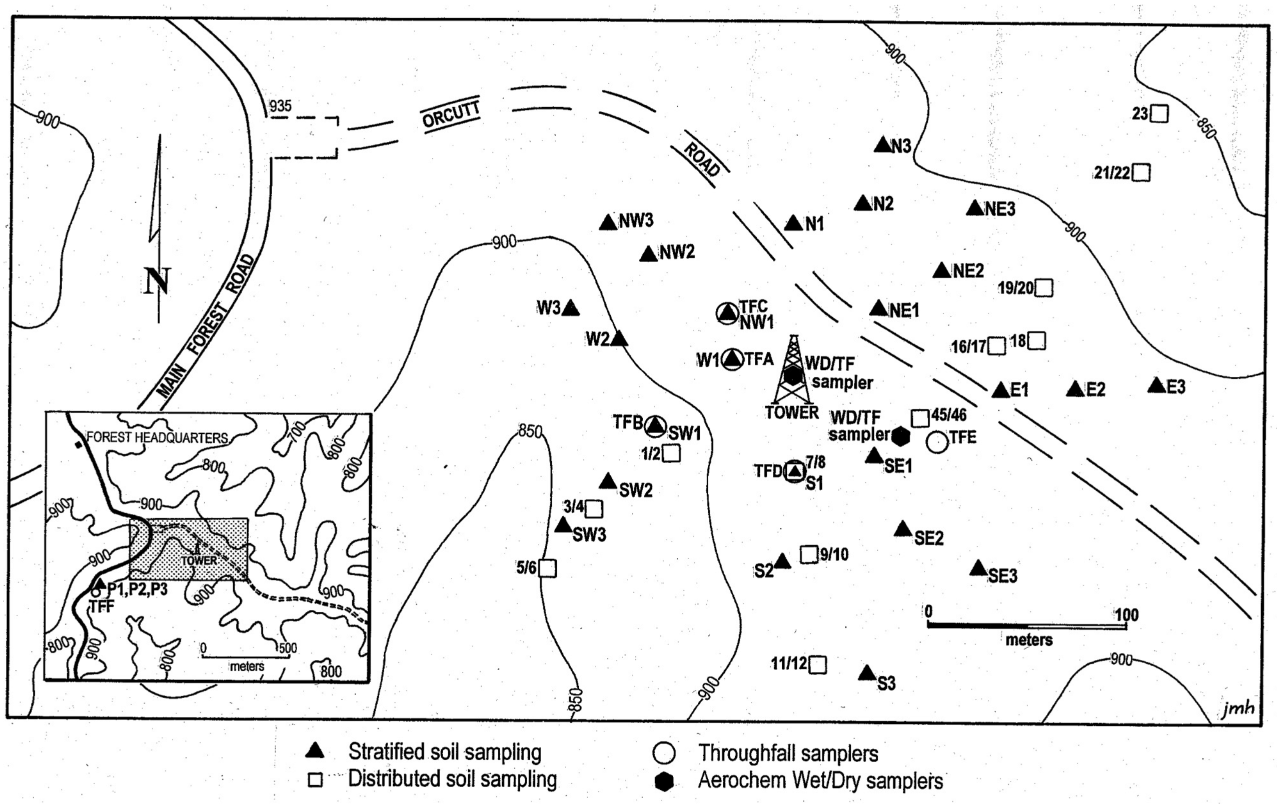

**FIGURE 2.** Location of soil and throughfall sampling at MMSF.

**TABLE 1**
**Instrumentation Details of Atmospheric Measurements at MMSF Conducted to Date**

| | Instrumentation | Sampling | Inlet Heights (m) | Notes |
|---|---|---|---|---|
| | | **Episodic Measurements** | | |
| $HNO_3$ | NaCl-coated denuders | 4 to 18 h integrated | 16.0, 27.4, 37.0, 44.0 | 3 to 14 samples, 2 to 4 blanks per height* |
| $HNO_3$ | Denuder-based REA system | 2 to 4 h integrated | 39.4 | 2 blanks per period* |
| $NH_3$ | Oxalic acid–coated denuders | 4 to 18 h integrated | 16.0, 27.4, 37.0, 44.0 | 3 to 14 samples, 2 to 4 blanks per height* |
| $NH_3$ | Wet effluent diffusion Denuders (WEDD)[17] | Continuous (2 min. av.) | 16.8/27.8, 37.2, 45.7 | |
| Particle size/ composition | MSP MOUDI 110 impactors | 8 to 24 h av. | 26.8, 40.0 | 11 stages. Dp 0.056 to >18 μm* |
| Particle size distribution | TSI APS 3320 | 10 s av. | 26.6/39.6 | Dp <0.524 to 19.81 μm |
| Wetness sensors | University of Bayreuth | 2 min. av. | 16.0 | 12 sensors, sunlit and shaded leaves |
| | | **Continuous Measurements** | | |
| NO/$NO_2$ | EcoPhys CLD770 + PLC760 | Continuous | 36.0, 40.0, 44.0 | |
| $O_3$ | Unisearch LOZ-3 | Continuous | 36.0, 40.0, 44.0 | |
| WD&TF | 2* Aerochem wet-dry | Weekly | 26.8, below canopy | * |
| Distributed TF | 18 purpose-built samplers | Weekly | 1 | * |
| SF | 3 purpose-built samplers | Weekly | | * |

* = Analysis by HPIC. The analytical precision as determined by replica sampling for the range of concentrations in the ambient samples are ±5% for $NH_4^+$ and ±10% for $NO_3^-$.

**TABLE 2**
**Mean pH, N Fluxes, and Sky View Factors at the Throughfall Measurement Clusters**

| | WD/TF | TFA | TFB | TFC | TFD | TFE | Pines |
|---|---|---|---|---|---|---|---|
| Mean pH | 5.2/6.1 | 5.8 | 5.8 | 5.7 | 5.6 | 5.7 | 5.1 |
| Mean $NO_3^-$ mg-N $m^{-2}$ $day^{-1}$ | 0.9/1.0 | 1.1 | 1.3 | 1.1 | 1.0 | 1.0 | 1.5 |
| Mean $NH_4^+$ mg-N $m^{-2}$ $day^{-1}$ | 2.2/1.1 | 1.3 | 1.4 | 1.1 | 1.6 | 1.6 | 1.5 |
| Dominant canopy species | SM/H | SM/B | SM/TP | SM | SM/H | SM | Pines |
| SVF (leaf off) | 0.500 | 0.502 | 0.457 | 0.488 | 0.518 | 0.498 | 0.346 |
| SVF (leaf on) | 0.175 | 0.203 | 0.181 | 0.167 | 0.181 | 0.155 | 0.175 |

*Notes:* Mean pH and N fluxes for the distributed sites reflect data collected: September 29, 2000–June 16, 2001. The data for the central WD/TF site reflect samples collected during April 13, 2000–June 16, 2001.
SM = sugar maple, H = hickory, TP = tulip poplar, B = beech.

friction velocity[20], and the resistance analogue[21]. The results indicate significant atmosphere-surface exchange of $HNO_3$, so a denuder-based $HNO_3$ relaxed eddy accumulation (REA) system has been constructed and was deployed during summer 2001. While nitric oxide (NO) and nitrogen dioxide ($NO_2$) are expected to be present at relatively high concentrations at MMSF, their low deposition velocities and uptake in the biosphere are anticipated to result in a small but seasonally persistent contribution to N deposition to a forest ecosystem. Hence, beginning in summer 2001, continuous measurements of NO and $NO_2$ commenced in combination with ozone ($O_3$) measurements to allow for correction of the derived fluxes for reactions within the triad $NO/NO_2/O_3$[22].

Direct measurement of particle dry deposition is confounded by the myriad of processes that determine the deposition and difficulties in sampling particle size (and composition) with sufficient resolution. Hence, the fluxes presented here are derived using size and chemically resolved particle measurements combined with modeled dry deposition velocities ($v_d$). Because modeling of particle $v_d$ is also uncertain particularly for particle diameters of 0.1 to 1.0 µm, three models[23,24,25] have been applied to quantify how this uncertainty is manifest in dry deposition estimates of particle-bound N calculated for MMSF. The modeled deposition fluxes differ by a factor of three for $NO_3^-$ fluxes and a factor of ten for $NH_4^+$, due to the concentration of $NH_4^+$ in the accumulation mode, where greatest uncertainties in $v_d$ are manifest (Fig. 3). During April 2000, two MOUDI impactors were deployed in gradient configuration (at 40 and 28 m) to experimentally determine $v_d$ for particles below 2-µm diameter from the observed difference in $NO_3^-$ concentrations under the assumption of diffusion-dominated transfer. The mean $v_d$ derived in this way (Fig. 3) shows closest agreement with the empirical model[23], and so fluxes calculated using $v_d$ from this model are presented here.

Micrometeorological techniques for flux estimation are reliant on the constant flux layer assumption. However, this assumption may not be applicable because of advection (due to horizontal concentration gradients) or storage effects (due to changes in the concentration within the air column), which are approximately proportional to the measurement height, and chemical reactions between the measurement height and the ground. In the flux calculations presented here, no correction has been applied to account for these effects, although there is some evidence of above-canopy chemical flux divergence in the $NH_3$ data[17]. Research into the validity of gradient approaches to determining dry deposition fluxes of passive tracers to a forest have determined mean enhancement factors (defined as the ratio of eddy-covariance-derived fluxes to fluxes derived using similarity theory) of $1.1 \pm 0.06$ and $1.24 \pm 0.07$ for a measurement height at approximately twice canopy height for pre- and

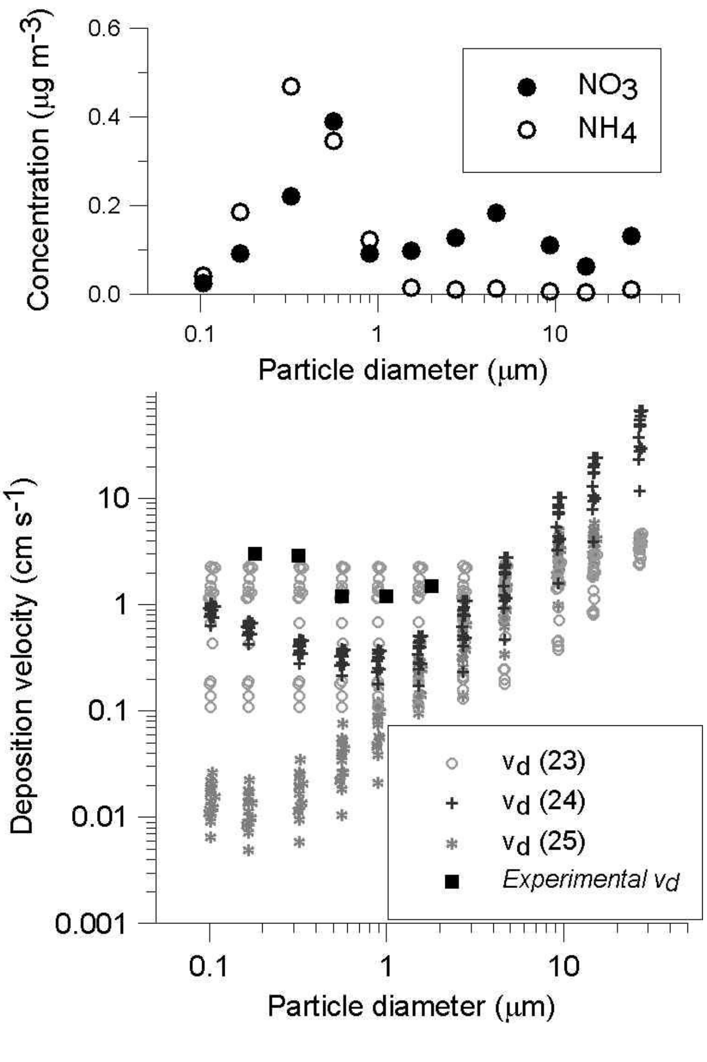

**FIGURE 3.** Mean size resolved $NH_4^+$ and $NO_3^-$ at MMSF during April–May 1998 (above) and the modeled $v_d$ from each of three models[23,24,25] for each sample during this experiment and particle diameter corresponding to the MOUDI cut-points (below). Also shown on the lower frame is the mean $v_d$ for particle diameters 0.1 to 2 µm experimentally determined for MMSF during April 2000.

postsenescence, respectively, and 1.6 ± 0.1 and 1.82 ± 0.11 for measurements conducted at 1.2 to 1.4 times canopy height[26]. Hence, although similarity theory can be applied for measurements taken at or above 1.6 times the canopy height, it should be acknowledged that the fluxes presented here may represent an underestimation of the actual dry deposition of N compounds.

To provide a preliminary index of the forest N status and response to atmospheric N addition, internal N cycling rates are being measured at MMSF as net N mineralization (the conversion of organic N to inorganic forms that are available for plant uptake) and net nitrification (the oxidation of $NH_4^+$ to $NO_3^-$, which is a mobile form of N, readily lost from ecosystems). *In situ*, net N mineralization and net nitrification rates are measured monthly during the growing season and overwinter at each site using the buried bag technique[27]. This technique measures the difference in inorganic N between initial and field-incubated soil samples. Soil sampling at MMSF comprises two sampling designs (Fig. 2):

- 24 plots oriented in 3 rings along 8 transects under sugar maples (and for comparison at a single site in a Red Pine Plantation). This sampling design was developed because sugar maples are an abundant canopy tree in MMSF and have a relatively large geographic range which would allow comparisons with other sugar maple–dominated deciduous forests in North America.
- 22 plots on 3 transects running north, south, and southwest of the tower, where annual NPP, litterfall, and other ecological measurements are conducted. This soil sampling scheme is designed to capture spatial variability and answer questions regarding mass balance of N cycling at the stand level.

As part of the ecological inventory[11], biomass litter and soil samples were taken from 22 nested plots. Foliage, branches, and bole wood samples were taken from dominant tree species. Foliage samples were taken from dominant understory vegetation and from litter. Soil was excavated in 20-cm increments from a 25- × 25-cm pit to a depth of 1 m or to bedrock. Litterfall was collected biweekly in two 0.5-$m^2$ mesh traps in each plot during each autumn. All samples were dried in a 65°C oven, ground to 100 mesh in a Wiley mill, and analyzed using PerkinElmer CHN and ICP analyzers. Additionally, in midsummer (August 15, 2000), leaves were removed from each of the 24 sugar maples below which N mineralization is measured. The leaves were ground through a fine mesh using a Wiley Mill, oven dried at 65°C, and their %N and %C measured using a CHN analyzer (PerkinElmer Model 2000).

## N FLUXES, POOLS, AND CYCLING

Total inorganic N wet deposition fluxes measured at MMSF during the growing season of 2000 are comparable to those measured at NADP sites located in Indiana (IN 22), Kentucky (KY 35), and Ohio (OH 09), but at MMSF the dominant form is $NH_4^+$, while the dominant ion is $NO_3^-$ at the NADP sites. Mean wet deposition fluxes at IN 22, OH 09, and KY 35 for the growing seasons (April to September, inclusive) of 1997–2000 are 1.6 to 2.2 mg-N $m^{-2}$ $day^{-1}$, while the median fluxes at MMSF based on 35 samples were 1.0 mg-N $m^{-2}$ $day^{-1}$ as $NO_3^-$ and 1.9 mg-N $m^{-2}$ $day^{-1}$ as $NH_4^+$. In accord with previous studies[28], the pH of throughfall at MMSF is predominantly higher than that in wet deposition, particularly during the leaf-on period, and significantly lower (paired t-test, 99% confidence level) under the coniferous canopy (Table 2).

Average dry deposition fluxes for the field experiments conducted during the growing seasons of 1998–2000 are $HNO_3$: 0.4 to 0.8 mg-N $m^{-2}$ $day^{-1}$, $NH_3$: 0.2 to 1.0 mg-N $m^{-2}$ $day^{-1}$; aerosol $NH_4^+$: 0.2 to 0.6 mg-N $m^{-2}$ $day^{-1}$; aerosol $NO_3^-$: 0.2 to 1.0 mg-N $m^{-2}$ $day^{-1}$, giving an average growing season total N dry deposition of 2.2 mg-N $m^{-2}$ $day^{-1}$ (excluding the contribution from NOx, organic N, and aged N compounds) which is the same magnitude the average growing season wet deposition of 2.9 mg-N $m^{-2}$ $day^{-1}$. For comparative purposes, N deposition of $HNO_3$ and fine particle $NH_4^+$ and $NO_3^-$ as measured at 12 sites across America in the Integrated Forest Study (IFS) ranged between 100 and 900 mol-N $ha^{-1}$ $year^{-1}$ (equivalent to an daily average flux of 0.4 to 3.6 mg-N $m^{-2}$). Typically the forest acts as a sink for $NH_3$ (representative daily flux 1.4 mg-N $m^{-2}$). However, inverted concentration gradients of $NH_3$ are occasionally observed during spring, resulting in an upward flux of nearly 0.2 mg-N $m^{-2}$ $h^{-1}$. These apparent upward fluxes of $NH_3$ may result from stomatal release. However, there is evidence that chemical flux divergence due to particle volatization may be contributing to the inverted gradients[17]. The results of field experiments further indicate a substantial fraction of reactive N is present in the particle phase during winter and spring (up to 80% of NHx and non-NOx total oxidized N); hence on the annual level, particle dry deposition is a nonnegligible contributor to the total dry deposition flux.

In accord with the results of the IFS[29], the average ratio of $NO_3^-$ in wet deposition to throughfall during the growing season is 1.8, while that for $NH_4^+$ is 3.7. This finding indicates considerable canopy retention of N, and preferential retention of the reduced form ($NH_4^+$). As in the IFS, the data collected during the 2000 growing season indicate approximately 40% of inorganic N deposited to the MMSF canopy is taken up by the canopy during the growing season (see below).

Mean annual total organic N mineralized to inorganic N under sugar maples was 104 µg N (SE ± 11.0) and 1580 µg N (SE ± 151) per g dry weight (DW) and ash-free dry weight (AFDW) soil (Table 3). Nitrification rates were extremely high, with over 90% of total N mineralized being converted to $NO_3$-N. The coefficients of variation for total N mineralization and nitrification were 49% on a DW soil basis and 45% on an AFDW basis. Thus these results indicate a highly nitrifying forest. The N mineralization rate for MMSF was 115 kg-N $ha^{-1}$ $year^{-1}$, and the nitrification rate was 107 kg-N $ha^{-1}$ $year^{-1}$. The growing season accounted for only 40% of the year but provided 67% of the total N mineralized. Of the total N mineralized during the growing season, 98% was transformed to $NO_3$-N, an exceptionally high proportion. The results of the distributed soil sampling conducted at MMSF were very similar to those under sugar maples. Annual net N mineralization rates were approximately 130 kg N $ha^{-1}$, with net nitrification rates ranging from 94 to 100% of total N mineralized in June and July, to 34 to 43% at the end of the growing season, with overwinter rates of 81%. Monthly net N mineralization rates exhibited significant spatial variability where rates were lower on the S-facing slope. The mineralization and nitrification rates derived for MMSF are similar to those derived for the Fernow Experimental Forest (Table 3), which is also a mixed

**TABLE 3A**
**Soil Sampling Results***

| | 10/29–12/11 | 12/11–04/05 | 4/05–5/11 | 5/11–6/18 | 6/18–7/17 | 7/17–8/15 | 8/15–9/29 | 9/29–11/02 | Ann. | GS |
|---|---|---|---|---|---|---|---|---|---|---|
| MMSF: Net N min. (kg N $ha^{-1}$ $interval^{-1}$) | 7.7 | 6.8 | 11 | 25.3 | 22 | 23.6 | 6.1 | 12.3 | 115 | 77 |
| MMSF: Net nitrification (kg $NO_3$-N $ha^{-1}$ $interval^{-1}$) | 5.7 | 6.5 | 6.5 | 24.8 | 18.5 | 25.9 | 6.5 | 12.4 | 107 | 76 |
| Extractable N (µg inorg. N /g DW soil) | 4.0 | 3.7 | 5.0 | 5.0 | 9.4 | 10.8 | 11.1 | 10.4 | | |
| Extractable $NO_3$-N (µg N /gDW soil) | 2.0 | 1.8 | 1.3 | 1.2 | 7.4 | 6.9 | 8.5 | 6.6 | | |
| | | | | **June** | **July** | **Aug.** | **Sept.** | **Oct.** | | |
| FEF: Net N min. (kg N $ha^{-1}$ $month^{-1}$) | | | | 30–32 | 9.5–12 | 17–18 | 11–15 | 4–5.5 | | |
| FEF: Net nitrification (kg $NO_3$-N $ha^{-1}$ $month^{-1}$) | | | | 19–27 | 13–15 | 17–18 | 7–12 | 4–5.5 | | |

* N mineralization and nitrification rates below sugar maples in MMSF vs. those from untreated stands in Fernow Experimental Forest (FEF)[30]. Also shown is extractable inorganic N below sugar maples at MMSF. Data presented for MMSF are for the period November 1999–October 2000.
GS = growing season rate computed as the total of total N produced from 5/11/00 to 9/29/00.

**TABLE 3B**
**N Soil Cycling Results from the Distributed Sampling at MMSF: June 1999–November 2000**

| | Jun. | Jul. | Au. | Sep. | Oct. | Winter | Apr. | May | Jun. | Jul. | Aug. Sep. | Oct. | Nov. | Ann. | GS Tot. |
|---|---|---|---|---|---|---|---|---|---|---|---|---|---|---|---|
| | | | 1999 | | | 99–00 | | | | 2000 | | | | | |
| Net N min.* | 23.9 | 10.5 | 18.7 | 13.2 | 27.0 | 5.3 | 9.8 | 21.2 | 21.8 | 14.2 | 18.8 | 11.6 | 6.3 | 130 | 97 |
| Net nitrification+ | 95 | 100 | 34 | 43 | 81 | 100 | 100 | 92 | 100 | 97 | 88 | 100 | 100 | | |

* = kg-N $ha^{-1}$ $period^{-1}$. + = % net N min. GS = growing season rate = total of N produced May–Sept., inclusive.

hardwood forest with significant presence of sugar maples, and which is showing evidence of N saturation[30].

Potassium chloride extractable soil $NH_4$-N and $NO_3$-N concentrations beneath the sugar maples in MMSF were highest during the growing season. The maximum extractable N and $NO_3^-$ occurred in August and were 3 and 4.7 times greater than their minima. The increase in inorganic N in the summer at MMSF may indicate that contributions of N from both microbial mineralization and atmospheric N deposition exceed the sink strength of plants for N in this forest. This hypothesis is to be addressed by adding lysimeters to the measurement suite in the upcoming year to quantify leaching losses.

A summary of %N in various ecosystem components is shown in Table 4. Mean N abundance in foliage of the six dominant tree species ranges between 1.9 and 2.7%, with average C:N ratios of 20–25:1. C:N ratios in total aboveground biomass of these species is approximately 205:1 (±45). Mean percent foliage N for the 24 sugar maples collected during summer 2000 was 2.16% (with a range of 1.81 to 2.59%), and the average C:N ratio was 21.99 (with a range of 17.81 to 25.62).

## LINKING N AND C CYCLES: A FIRST ESTIMATE OF C SEQUESTRATION ENHANCEMENT DUE TO N FLUXES

The measurements presented here reflect a temporally limited data set. Nevertheless, an index of the perturbation of the MMSF ecosystems N cycle can be derived by examining the relative

**TABLE 4**
**Mean % N* for Various Ecosystem Components**

| CR1 | CR2 | CR3 | Duff | FR1 | FR2 | FR3 | H | LL | S | SB | T |
|---|---|---|---|---|---|---|---|---|---|---|---|
| 16 | 14 | 11 | 16 | 16 | 16 | 15 | 11 | 16 | 11 | 12 | 15 |
| 0.86 | 0.73 | 0.57 | 0.96 | 1.0 | 0.69 | 0.70 | 2.36 | 1.46 | 1.66 | 0.61 | 0.69 |

* Second line indicates sample number.
CR = coarse roots, 1 = 0 to 20 cm, 2 = 20 to 40 cm, 3 = 40 to 60 cm; Duff = partly decayed organic matter; FR = fine roots, 1 = 0 to 20 cm, 2 = 20 to 40 cm, 3 = 40 to 60 cm; H = herbaceous; LL = leaf litter; S = seedings; SB = small branches; T = twigs.

rates of root pathway uptake vs. direct canopy uptake[31]. An accurate assessment of canopy uptake of atmospheric N (net canopy exchange, or NCE) requires measurement of N-fluxes in wet deposition (WD), dry deposition (DD), stemflow (SF) and throughfall (TF):

$$\begin{aligned} NCE = & DD(NH_4\text{-}N + NO_3\text{-}N + DON\text{-}N + NO_{x/z}\text{-}N) \\ & + WD(NH_4\text{-}N + NO_3\text{-}N + DON\text{-}N) \\ & - TF(NH_4\text{-}N + NO_3\text{-}N + DON\text{-}N) \\ & - SF(NH_4\text{-}N + NO_3\text{-}N + DON\text{-}N) \end{aligned}$$

However, first estimates derived from measurements of DD, WD, and TF of inorganic N conducted to date at MMSF are –4.4 kg-N $ha^{-1}$ for reduced inorganic N and –2.9 kg-N $ha^{-1}$ for oxidized inorganic N to give a total growing season NCE of –7.3 kg-N $ha^{-1}$. These values indicate the canopy is taking up approximately 7 kg-N $ha^{-1}$ directly from the atmosphere during the growing season. The NCE for the entire year is approximately 9 to 10 kg-N $ha^{-1}$, based on WD and TF data collected between April 2000 and March 2001. These values are substantially smaller than the average litterfall rate of 64 kg-N $ha^{-1}$ and total growing season mineralization of approximately 100 kg-N $ha^{-1}$. However, if one assumes that C uptake by the canopy is solely limited by N[31], then for an average foliar C:N ratio of 20 to 25 (as measured at MMSF), this implies additional C uptake of 180 to 250 kg-C $ha^{-1}$ due to the net canopy uptake of N compounds, which is approximately 10% of current C storage (see Table 5).

It should be noted that multiple factors beyond those described herein complicate consideration of N-C coupling under future environmental scenarios. It is possible, indeed probable, that coupling of C and N cycles may alter under changed environmental conditions. The decay processes that release C and N from plant litter to the soil are strongly interlinked[32,33], but while increasing temperature increases microbial activity, and hence $CO_2$ emission[34], adding N to decomposing organic matter may increase or decrease decomposition rates[35]. Additionally, experimental $CO_2$ enhancement studies have shown that while increased $CO_2$ availability enhanced plant N uptake, it reduced available soil N and hence enhanced N limitations on microbes and slowed microbial decomposition[36]. Further recent research has revealed significant interspecies variations in N uptake response to elevated $CO_2$[37].

Plans are in place to measure leaching and dinitrificiation losses to allow closure of the N budget for the MMSF ecosystem and determine whether this forest is approaching N saturation. Also, samples will be collected and analyzed to quantify total

**TABLE 5**
**Best Estimate N Cycling Rates for MMSF**
**Compared to Those for Howland Forest Spruce Fir Site[31]**

| | MMSF | Howland Forest[31] |
|---|---|---|
| Mineralization (kg-N $ha^{-1}$ $year^{-1}$) | 115 – 130 | 20.5 |
| Litterfall (kg-N $ha^{-1}$ $year^{-1}$) | 64 | 12.2 |
| NCE (kg-N $ha^{-1}$ $year^{-1}$) | 9 – 10 | 3.6 |
| Aboveground C:N ratio | 205 | 250 – 270 |
| Foliar C:N ratio | 20 – 25 | Not given |
| Possible C storage enhancement (kg-C $ha^{-1}$ $year^{-1}$) | 200 – 2050* | 250 – 1350 |
| C sequestration (kg-C $ha^{-1}$ $year^{-1}$) | 2400 | 2100 |

*Note:* The NCE values quoted for Howland Forest were calculated using weekly air concentrations.

* Lower bound assumes C uptake in C:N ratio of foliage; upper bound assumes C uptake in C:N ratio of aboveground biomass.

DON in precipitation, throughfall, and stemflow. It is likely that these data will show DON makes a minor contribution to N in precipitation and that the NCE for organic N is negative, indicating canopy release of organic N, causing a reduction in total N NCE.

## ACKNOWLEDGEMENTS

Fifty percent of this research was funded by the National Institute for Global Environmental Change through the U.S. Department of Energy (Cooperative Agreement No. DE-FC03-90ER61010). The remaining financial support was supplied by the National Science Foundation (grant ATM-9977281). Any opinions, findings, and conclusions or recommendations expressed in this publication are those of the authors and do not necessarily reflect the views of the U.S. DOE or NSF.

## References

1. IGBP (1998) The terrestrial carbon cycle: implications for the Kyoto Protocol. *Science* **280,** 1393–1394.
2. Vitousek, P.M. and Howarth, R.W. (1991) Nitrogen limitation on land and in the sea: how can it occur? *Biogeochemistry* **13,** 87–115.
3. Galloway, J.N. (1998) The global nitrogen cycle: changes and consequences. *Environ. Pollut.* **102**(Suppl. 1), 15–14.
4. Melillo, J.M. (1981) Nitrogen cycling in deciduous forests. *Ecol. Bull.* **33,** 427–442.
5. Sievering, H. (1999) Nitrogen deposition and carbon sequestration. *Nature* **400,** 629–630.
6. Nadelhoffer, K., Emmett, B., Gundersen, P., Kjoenaas, O., Koopmans, C., Schlepp, P., Tietema, A., and Wright, R. (1999) Nitrogen deposition makes a minor contribution to carbon sequestration in temperate forests. *Nature* **398,** 145–147.
7. IPCC (Intergovernmental Panel on Climate Change). (1996) *Climate Change 1995*. Cambridge University Press, London. pp. 572.
8. Beier, C. and Eckersten, H. (1998) Modelling the effects of nitrogen addition on soil nitrogen status and nitrogen uptake in a Norway spruce stand in Denmark. In *Nitrogen, the Confer-N-s*. Elsevier, Noordwijkerhout, The Netherlands. pp. 409–414.
9. Schjoerring, J.K. (1997) Plant-atmosphere ammonia exchange: quantification, physiological regulation and interactions with environmental factors. In Plant Nutrition and Soil Fertility Laboratory. Department of Agricultural Sciences. Royal Veterinary and Agricultural University, Copenhagen.
10. Schmid, H.P., Grimmond, C.S.B., Cropley, F., Offerle, B., and Su, H.-B. (2000) Measurements of $CO_2$ and energy fluxes over a mixed hardwood forest in the midwestern United States. *Agric. For. Meteorol.* **103,** 357–374.
11. Ehman, J., Schmid, H., Grimmond, C., Hanson, P., Randolph, J., and Cropley, F. (1999) A preliminary inter-comparison of micrometeorological and ecological estimates of carbon sequestration in a mid-latitude deciduous forest. *Proc. 15th Intl. Conf. Biometeorology*. Sydney, Australia. pp. 6.
12. Cornell, S., Rendell, A., and Jickells, T. (1995) Atmospheric inputs of dissolved organic nitrogen to the oceans. *Nature* **376,** 243–246.
13. Sirois, A., Vet, R., and Lamb, D. (2000) A comparison of the precipitation chemistry measurements obtained by the CAPMON and NADP/NTN networks. *Environ. Monit. Assess.* **62,** 273–303.
14. Claassen, H.C. and Halm, D.R. (1995) A possible deficiency in estimates of wet deposition obtained from data generated by the NADP/NTN network. *Atmos. Environ.* **29,** 437–448.
15. Houle, D., Ouimet, R., Paquin, R., and Laflamme, J.G. (1999) Determination of sample size for estimating ion throughfall deposition under a mixed hardwood forest at the Lake Clair Watershed (Duchesnay, Quebec). *Can. J. For. Res.* **29,** 1935–1943.
16. Beier, C., Rasmussen, L., Pilegaard, K., Ambus, P., Mikkelsen, T., Jensen, N.O., Kjoller, A., Prieme, A., and Ladekarl, U.L. (2001) Fluxes of $NO_3^-$, $NH_4^+$, NO, $NO_2$, and $N_2O$ in an old Danish Beech forest. *Water Air Soil Pollut.: Focus* **1**, 187–195.
17. Pryor, S.C., Barthelmie, R.J., Sørensen, L., and Jensen, B. (2001) Ammonia concentrations and fluxes over a forest in the midwestern USA. *Atmos. Environ.,* **35,** 5645–5656.
18. Meyers, T.P., Hall, M.E., Lindberg, S.E., and Kim, K. (1996) Use of the modified Bowen-ratio technique to measure fluxes of trace gases. *Atmos. Environ.* **30,** 3321–3329.
19. Muller, H., Kramm, G., Meixner, F., Dollard, G., Fowler, D., and Possanzini, M. (1993) Determination of $HNO_3$ dry deposition by modified Bowen ratio and aerodynamic profile techniques. *Tellus* **45B,** 346–367.
20. Andersen, H., Hovmand, M., Hummelshoj, P., and Jensen, N.O. (1993) Measurements of ammonia flux to a spruce stand in Denmark. *Atmos. Environ.* **27A,** 189–202.
21. Hicks, B.B., Baldocchi, D.D., Meyers, T.P., Hosker, R.P., and Matt, D.R. (1987) A preliminary multiple resistance routine for deriving dry deposition velocities. *Water Air Soil Pollut.* **36,** 311–330.
22. De Arellano, J. and Duynkerke, P. (1992) The influence of chemistry on the flux-gradient relationships in the NO-$O_3$-$NO_2$ system. *Bound.-Layer Meteorol.* **61,** 375–387.
23. Erisman, J., Draaijers, G., Duyzer, J., Hofschreuder, P., Van Leeuwen, N., Romer, F., Ruijgrok, W., Wyers, P., and Gallagher, M. (1997) Particle deposition to forests: summary of results and application. *Atmos. Environ.* **31,** 321–332.
24. Wiman, B., Agren, G., and Lannefors, H. (1985) Aerosol concentration profiles within a mature coniferous forest: model versus field results. *Atmos. Environ.* **19**, 363–367.
25. Slinn, W. (1982) Predictions for particle deposition to vegetative canopies. *Atmos. Environ.* **16,** 1785–1794.
26. Simpson, I.J., Thurtell, G.W., Neumann, H.H., Den Hartog, G., and Edwards, G.C. (1998) The validity of similarity theory in the roughness sublayer above forests. *Bound.-Layer Meteorol.* **87,** 69–99.
27. Gordon, A.M., Tallas, M., and Van Cleve, K. (1987) Soil incubation in polyethylene bags: effect of bag thickness and temperature on nitrogen transformations and $CO_2$ permeability. *Can. J. Soil Sci.* **67**, 65–75.
28. Parker, G.G. (1990) Evaluation of dry deposition, pollutant damage, and forest health with throughfall studies. In *Mechanisms of Forest Response to Acidic Deposition*, Lucier, A.A. and Haines, S.G., Eds. Springer-Verlag, New York. pp. 245.
29. Cole, D.W. (1992) Nitrogen chemistry, deposition and cycling in forests. In *Atmospheric Deposition and Forest Nutrient Cycling: A Synthesis of the Integrated Forest Study*. Johnson, D.W. and Lindberg, S.E., Eds. Springer-Verlag, New York. pp. 707.
30. Gilliam, F.S., Adams, M.B., and Yurish, B.M. (1996) Ecosystem nutrient responses to chronic nitrogen input at Fernow Experimental Forest, West Virginia. *Can. J. For. Res.* **26,** 196–205.
31. Sievering, H., Fernandez, I., Lee, J., Hom, J., and Rustad, L. (2000) Forest canopy uptake of atmospheric nitrogen deposition at eastern U.S. conifer sites: carbon storage implications? *Glob. Biogeochem. Cy.* **14,** 1153–1160.
32. Melillo, J.M., Aber, J.D., Linkins, A.E., Ricca, A., Fry, B., and Nadelhoffer, K.J. (1989) Carbon and nitrogen dynamics along the decay continuum: plant litter to soil organic matter. *Plant Soil* **115,** 189–198.
33. Aber, J.D., Melillo, J.M., and McLaugherty, C.A. (1990) Predicting long-term patterns of mass loss, nitrogen dynamics and soil organic matter formation from initial fine litter chemistry in temperate forest ecosystems. *Can. J. Bot.* **68,** 2201–2208.
34. Davidson, E.A. (1995) Linkages between carbon and nitrogen cycling and their implications for storage of carbon in terrestrial ecosystems. In *Biotic Feedbacks in the Global Climatic System*. Woodwell, G.M. and MacKenzie, F.T., Eds. Oxford University Press, New York. pp. 219–230.
35. Carreiro, M.M., Sinsabaugh, R.L., Repert, D.A., and Parkhurst, D.E. (2000) Microbial enzyme shifts explain litter decay responses to simulated nitrogen deposition. *Ecology* **81,** 2359–2365.
36. Hu, S., Chapin III, F.S., Firestone, M.K., Field, C.B., and Chiariello, N.R. (2001) Nitrogen limitation of microbial decomposition in grassland under elevated $CO_2$. *Nature* **409,** 188–191.
37. Zerihun, A. and BassiriRad, H. (2001) Interspecies variation in nitrogen uptake kinetic responses of temperate forest species to elevated $CO_2$: potential causes and consequences. *Glob. Change Biol.* **7,** 211–222.

| | | |
|---|---|---|
| Received: | July | 21, 2001 |
| Revised: | November | 1, 2001 |
| Accepted: | November | 9, 2001 |
| Published: | December | 5, 2001 |
